# Legacy effects of herbicides on soil nitrifying guilds exposed to drought

**DOI:** 10.1093/femsec/fiag038

**Published:** 2026-04-16

**Authors:** Laura J Müller, Aurélien Saghaï, Christopher M Jones, Sara Hallin

**Affiliations:** Department of Forest Mycology and Plant Pathology, Swedish University of Agricultural Sciences, 75651 Uppsala, Sweden; Department of Forest Mycology and Plant Pathology, Swedish University of Agricultural Sciences, 75651 Uppsala, Sweden; Department of Forest Mycology and Plant Pathology, Swedish University of Agricultural Sciences, 75651 Uppsala, Sweden; Department of Forest Mycology and Plant Pathology, Swedish University of Agricultural Sciences, 75651 Uppsala, Sweden

**Keywords:** nitrification, soil, microbial ecotoxicology, multiple stressors, ammonia oxidizing micro-organisms, nitrite oxidizing bacteria

## Abstract

Micro-organisms are essential for the functioning of agricultural soils but face increasing stress due to pollution and climate change. However, direct and legacy effects of agrochemicals on non-target micro-organisms are poorly considered in environmental risk assessment. Here, we set up a two-phase microcosm experiment to assess the effect of the herbicides clopyralid, metribuzin, and tembotrione (phase 1) on the abundance and activity of ammonia and nitrite oxidizing micro-organisms involved in nitrification, a key step in soil N cycling, and how it influences their response to subsequent drying and rewetting stress (phase 2). Pesticide exposure in phase 1 did not affect the nitrifying guilds and nitrification activity. By contrast, drying-rewetting affected the abundance of the different guilds, with ammonia-oxidizing archaea and *Nitrospira*-type nitrite oxidizers showing low resistance to rewetting, but with minor differences between herbicide-treated and no-herbicide treated soils. Legacy effects of herbicide exposure were instead captured by the soil nitrate pools, where differences between droughted and control soils appeared larger in the no-herbicide than in the herbicide-treated soils, potentially indicating differences in drought-coping strategies depending on prior stress exposure. Our results highlight that multiple stressor scenarios can reveal effects not captured by end-point measurements in risk assessment procedures.

## Teaser

Herbicides exhibited only minimal effects on abundance and activity of nitrifying micro-organisms, but potentially modified “generally stronger” subsequent drought effects, highlighting the importance of considering multiple stressor scenarios.

## Introduction

Pesticides are an integral part of modern agriculture and contribute to increased crop yields and yield stability by combating pathogens and crop pests, including weeds. At the same time, pesticides represent a threat to the ecological integrity of soils due to potential harmful effects on non-target micro-organisms (Hernández et al. 2011, Puglisi et al. 2012, Thiour-Mauprivez et al. 2019), which play key roles in agroecosystems by contributing to decomposition, nutrient cycling and plant growth (Brussaard 2012, Hartmann and Six 2023). Toxic effects on soil micro-organisms can be both direct and indirect (Meyer et al. 2024) and depend on the pesticide’s mode of action (Karpouzas et al. 2022). Current risk assessment procedures of pesticide toxicity in soils implemented in the European Union rely on an outdated nitrogen (N) transformation test, which can only be considered a rough estimate due to low sensitivity (Martin-Laurent et al. 2013, Pedrinho et al. 2024). In 2017, the European Food Safety Authority (EFSA) emphasized the need for novel tests to assess the toxicity of pesticides on soil micro-organisms (EFSA PPR Panel 2017). Tests targeting soil microbial function and diversity as a means to assess soil health are scientifically established and widely used in research (Ritz et al. 2009, Stone et al. 2016, Schloter et al. 2017), including quantitative PCR of microbial functional genes (Jia et al. 2025). However, challenges remain in implementing these methods in a risk-assessment framework, such as harmonization and standardization of protocols and a need for agreement on threshold values (Philippot et al. 2012, Thiour-Mauprivez et al. 2019).

Ammonia oxidizing archaea (AOA) and bacteria (AOB), performing the first step of nitrification in the N cycle (Kuypers et al. 2018), have been suggested as relevant microbial indicators of soil functioning and toxicity of pesticides and pollutants (Wessén and Hallin 2011, EFSA PPR Panel 2017) due to their sensitivity to external perturbations (Pereira e Silva et al. 2013), narrow phylogenetic breadth (Purkhold et al. 2003, Alves et al. 2018), and the availability of tools to measure their activity, abundance, and diversity (Pell et al. 1998, Nicol and Prosser 2011, Vasileiadis et al. 2018). Studies on the impact of herbicides on ammonia oxidizing isolates or communities show varying (e.g. Bachtsevani 2024, Karas et al. 2018) to limited effects (e.g. Thiour-Mauprivez et al. 2021, Sim et al. 2022). Comparatively few studies have examined the effects of herbicides or other pollutants on nitrite oxidizing communities (Fang et al. 2018, 2019, Rijk et al. 2023) performing the second step of nitrification, by oxidizing nitrite to nitrate, in soil mainly represented by the lineages *Nitrobacter* (NIB) and *Nitrospira* (NIS). Consequently, less is known about their relevance as indicators in risk assessment. Most ecotoxicological tests are typically performed under a single environmental condition, even though organisms in natural systems are simultaneously exposed to multiple stressors. Interaction effects between pollutants and other stressors are therefore increasingly recognized, including natural environmental stress due to sub-optimal conditions for growth (Holmstrup et al. 2010). One particularly relevant stressor in the context of climate change is drought. Dry and wet spells cause strong fluctuations in soil water potential (Schimel 2018), including changes in osmotic pressure and resource availability (Birch 1958). These conditions can alter the composition and activity of nitrifying communities (Stark and Firestone 1995, Bello et al. 2019, Bintarti et al. 2025, Müller et al. 2025), where differences between the functional guilds have often been attributed to differences in niche preferences, particularly their substrate affinities. AOA and NIS generally show higher substrate affinities than AOB and NIB, respectively (e.g. Verhamme et al. 2011, Sterngren et al. 2015, Nowka et al. 2015, Kits et al. 2017, Qin et al. 2024), resulting in stronger impacts on AOA and NIS during drying-rewetting stress and a potential destabilization of the shared nitrification pathway (Müller et al. 2025). Interactions between stressors are complex (Holmstrup et al. 2010, Galic et al. 2018) and knowledge about the resistance and resilience of nitrifying communities to multiple stressors is scarce. Addressing this knowledge gap is particularly relevant for improving the ecological realism of environmental risk assessment in soil.

**Figure 1:**
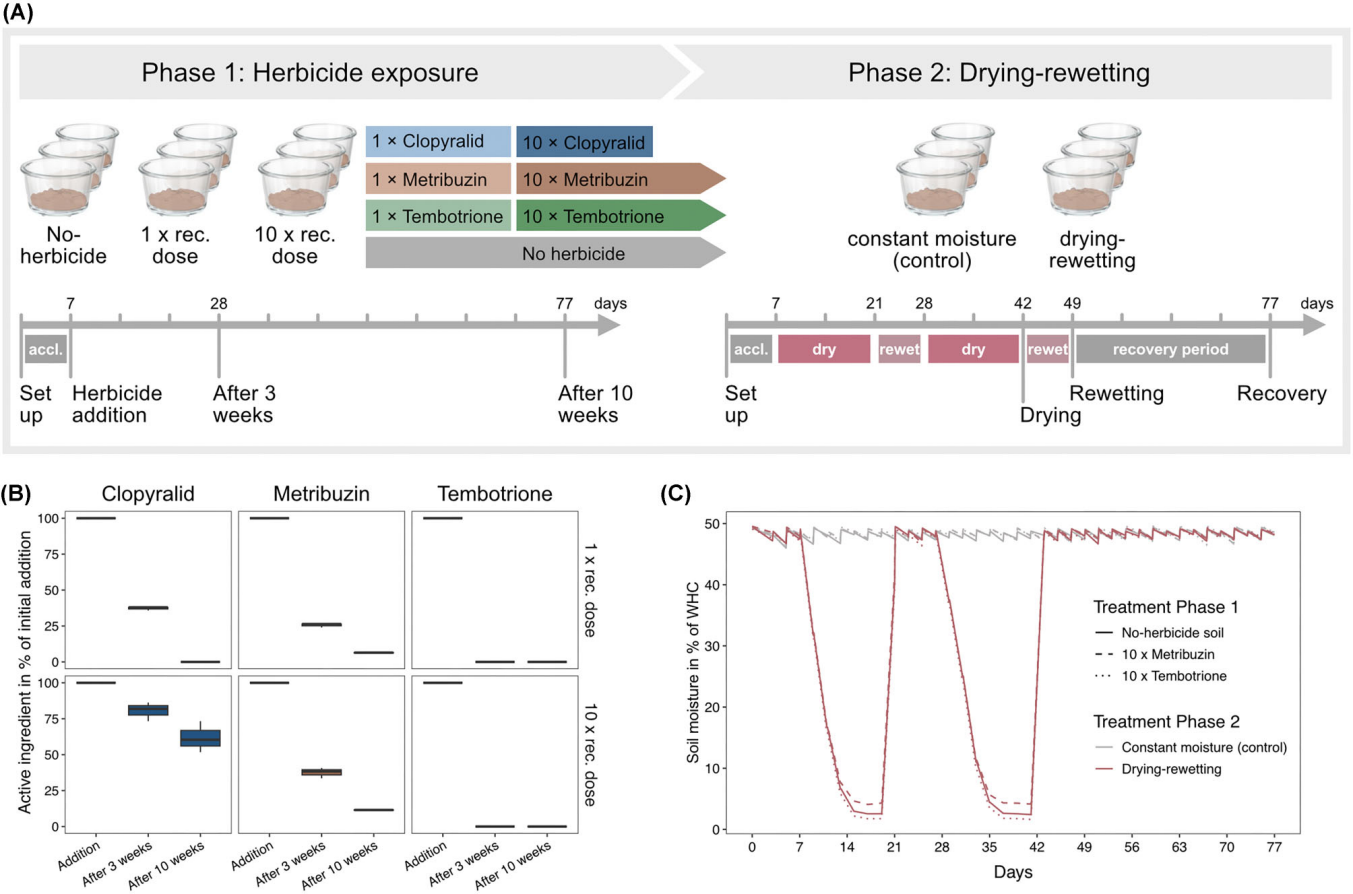
Overview of the microcosm experiments. A) Experimental design in phase 1 (herbicide application) and 2 (drying-rewetting). Phase 1 consists of seven treatments (herbicides clopyralid, metribuzin and tembotrione added at 1 × or 10 × the recommended dose and a no-herbicide control) applied after a 7-day acclimatization phase (“accl.”) with destructive sampling at three and ten weeks after herbicide addition. In phase 2, three treatments from phase 1 (no-herbide, 10 × metribuzin, 10 × tembotrione) were subjected to two drying-rewetting cycles or held at constant moisture over a period of 77 days, following an initial 7-day acclimatization phase (“accl.”). Pots were destructively sampled at the end of the second drought (“drying”), one week after rewetting (“rewetting”) and at the end of the recovery period (“recovery”). B) Remaining active ingredient three and ten weeks after addition to microcosms in phase 1, expressed as the percentage of active ingredient added initially. Measures below the detection limit of 0.050 mg kg^−1^ were set to zero. C) Changes in soil moisture in phase 2 microcosms throughout the period corresponding to the timeline in panel A. In the control pots, soil was kept at a constant soil moisture, ranging between 45% and 50% water holding capacity (WHC). Soil moisture was monitored and adjusted every two days. Line type represents the treatment in phase 1 and colour the treatment in phase 2.

In this study, we first assessed the effect of three herbicides with different modes of action (clopyralid, metribuzin, tembotrione; Table 1) on the abundance of canonical ammonia and nitrite oxidizers in soil, along with concomitant changes in soil ammonium and nitrate pools. We subsequently examined the resistance and resilience of these guilds to a second stressor, using drying-rewetting as an example. We set up a microcosm experiment with two phases (Fig. 1): in phase 1, herbicides were applied at either 1 × or 10 × the recommended dose according to EFSA. In phase 2, soil incubated with 10 × metribuzin, 10 × tembotrione, and the no-herbicide control soil were subjected to two drying-rewetting cycles to determine legacy effects of the herbicides on the resistance of nitrifiers to the secondary stressor, followed by a recovery phase to assess effects on their resilience. We hypothesized that (i) the three herbicides will exhibit distinct effects on nitrifier abundance and concomitant changes in ammonium and nitrate pools due to differences in the herbicides’ chemical composition, mode of action, and dose, (ii) subsequent drying and rewetting will differentially alter nitrifier abundances due to the variation in niche preferences among the guilds based on apparent substrate affinities (Kits et al. 2017), with AOA and NIS assumed to be more affected than AOB and NIB by the expected increase in nutrient content following rewetting, and (iii) nitrifying guilds negatively affected by the herbicide treatments will be more sensitive to additional stress and thus respond stronger to drying-rewetting than those unaffected from herbicides in phase 1.

**Table 1:**
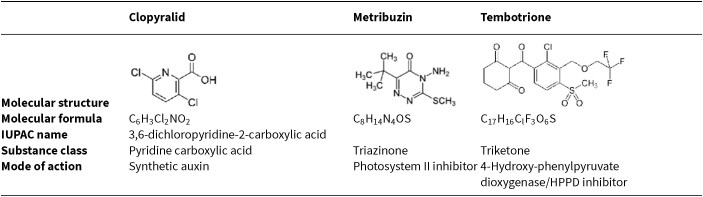
Characteristics of the three herbicides used in this study.

## Materials and methods

### Soil sampling and properties

Soil was collected (5–20 cm depth) in June 2021 from an agricultural field in Schnega, Germany (52°54′16.8“N, 10°49′54.9″E), homogenized, sieved (2 mm), and stored at −20°C until the experiment started. According to the USDA classification system, the soil is a loamy sand (0% clay, 25% silt, 75% sand, determined by PARIO method) with a pH_(H2O)_ of 5.4 and contained 1.10% total C (all organic), and 0.09% total N. Soil properties were determined at the Soil and Plant Laboratory (Swedish University of Agricultural Sciences, Uppsala, Sweden). Soil water content was measured as the weight difference before and after drying ∼5 g of soil at 105°C for 24 h in duplicates. Maximum water holding capacity (WHC) was estimated by determining gravimetric water content after the soil was soaked in water overnight, then drained for 5 h.

### Experimental design

In phase 1, the soil was thawed at 4°C for 36 h and left at room temperature for 24 h before the experiment was established. Round glass pots with an inner diameter of 12.5 cm were filled with 200 g fresh weight (FW) soil set at 45%–50% WHC, corresponding to a dry weight (DW) of 172 g. The microcosms did not allow for leaching of herbicides and were covered with sterile cotton cloth and aluminium foil to reduce evaporation while allowing soil aeration. They were placed in the dark in a climate chamber (20°C, 60% relative humidity; Fig. 1A). After one week of acclimatization, pesticides as pure active ingredient (a.i.) in aqueous solution or an equivalent amount of water (“no-herbicide soil”) were added and homogenized with the soil. Doses corresponded to 1 × or 10 × the recommended maximum annual dose following the EFSA guidelines, meaning 0.300 kg a.i. ha^−1^ clopyralid (EFSA Scientific Report 2005), 0.540 kg a.i. ha^−1^ metribuzin (EFSA 2019), and 0.100 kg a.i. ha^−1^ tembotrione (EFSA 2013). Considering a soil depth of 10 cm, this resulted in 0.232 and 2.32 mg a.i. kg^−1^ FW soil for clopyralid, 0.417 and 4.17 mg a.i. kg^−1^ FW soil for metribuzin, and 0.077 and 0.77 mg a.i. kg^−1^ FW soil for tembotrione in the 1 × and 10 × dose, respectively, in the microcosms. The experiment was set up to test toxicity effects on non-target soil organisms *in situ* rather than a realistic field scenario of pesticide spraying.

Clopyralid is a pre- and post-emergence herbicide applied in a range of crops, such as soy and potato, to control grasses and broadleaved weeds, and tembotrione is a post-emergence herbicide commonly used in maize to control similar types of weeds (Lewis et al. 2016). Metribuzin controls weeds, for example in potato, but the approval in the EU has recently not been renewed due to concerns for human health and a risk to bees (European Commission 2024).

In total, 108 microcosms were set up to allow for destructive sampling throughout phase 1 and for setting up phase 2 of the experiment. The soil moisture was monitored every second day by weighing the microcosms and sterile water was carefully pipetted on the soil surface when necessary to maintain a WHC of 45%–50% (Fig. 1A). There was no additional N input during or between the experimental phases. Triplicate microcosms for each treatment were destructively sampled three and 10 weeks (21 and 70 days) after pesticide addition and stored at −20°C until further analysis. Soil from the remaining microcosms was homogenized for each treatment and stored at −20°C until the setup of phase 2. Despite potential effects of freezing-thawing cycles, this step was necessary to avoid continued microbial activity and potential prolongation of recovery between experimental phases.

In phase 2, soils treated with only water (no-herbicide), 10 × metribuzin, and 10 × tembotrione were subjected to drought (Fig. 1A). Metribuzin is less studied than the other herbicides and tembotrione affects the 4-hydroxyphenylpyruvate dioxygenase (HPPD) enzyme, which is known to be present in micro-organisms (Thiour-Mauprivez et al. 2020). After seven days of acclimatization, soils were subjected to two drying-rewetting cycles, each consisting of a two-week drought period followed by rewetting and one week at 45%–50% WHC. The two cycles were followed by a final recovery period of four weeks. Control soils were kept at 45%–50% WHC throughout the experiment. Dry conditions were established by removing the aluminium foil cover and terminating soil moisture adjustment, leading to a decrease in soil moisture to an average of 4 (10 × metribuzin soil), 3 (no-herbicide soil), and 2 (10 × tembotrione) % WHC on day 21 and 42 (Fig. 1C). Microcosms were destructively sampled on day 42 (“drying”), after rewetting on day 49 (“rewetting”), and after the final recovery period on day 77 (“recovery”).

### Herbicide content in soil

Herbicide content (i.e. the active ingredient) in the soil was measured at both timepoints (Fig. 1B) by Eurofins Food & Feed Testing Sweden AB (Lidköping, Sweden). In brief, herbicides were extracted compound-specific from 5 g dw soil. Metribuzin was extracted using 5 ml ultrapure water, 5 ml acetone, 5 ml n-hexane, and NaCl (30 min), followed by centrifugation and analysis of the supernatant via GC-MS/MS. Tembotrione was extracted with 20 ml acetone (60 min) followed by centrifugation and evaporation to dryness, then re-dissolved in methanol and diluted with water. The membrane-filtered extract was then analyzed by LC-MS/MS. Clopyralid extraction and analysis followed the same procedure as tembotrione, except that soils were acidified with 500 µl concentrated HCl prior to acetone extraction.

### Measurement of soil ammonium and nitrate

To assess ammonium and nitrate content, extracts were prepared by shaking soil in 2 M potassium chloride (1 : 5 ratio) in 50 ml Falcon tubes on a horizontal shaker (1 h, 300 r/m) followed by centrifugation (5 min, 3500 *g*). The supernatant was subsequently filtered through Munktell 00H filter paper (Ahlstrom, Helsinki, Finland) and stored for a maximum of 4 days at 4°C until analysis on a segmented flow analyzer (AutoAnalyzer 500, SEAL Analytical, Inc., Mequon, Wisconsin, US).

### DNA extraction and quantification of *amoA* and *nxrB*

DNA was extracted with the NucleoSpin Soil kit (Macherey-Nagel, Düren, Germany) according to the manufacturer’s instructions using 0.4 g FW soil. DNA quality was validated by NanoDrop™ measurements (Thermo Fisher Scientific, Waltham, Massachusetts, US) and agarose gel electrophoresis before quantification with a Qubit® fluorometer (Thermo Fisher Scientific). To test for potential inhibition of PCR reactions by co-extracted contaminants, a known amount of pGEM-T plasmid (Promega, Madison, WI, USA) was amplified with plasmid-specific T7/SP6 primers and 2 ng of soil DNA or non-template controls. With this amount of DNA, no inhibition was detected. The abundance of total archaea and bacteria (16S rRNA gene), AOA (*amoA*), AOB (*amoA*), NIB (*nxrB*), and NIS (*nxrB*) were determined by real-time quantitative PCR (qPCR) using specific primers for the marker genes of each group. All reactions were performed in separate duplicate runs using 2 ng DNA in a reaction volume of 15 µl containing 1× mastermix and 1 µg/µl bovine serum albumin. The 16S rRNA gene was quantified using the iQ^TM^ SYBR Green Supermix (Bio-Rad, Hercules, California, USA) on a CFX384™ Real-Time System (Bio-Rad) and the genes involved in nitrification using Takyon™ Low ROX SYBR 2X MasterMix blue dTTP (Eurogentec, Seraing, Belgium) on a ViiA7 instrument (Life Technologies, Carlsbad, CA, USA). Efficiency ranged from 86% to 90%. Standard curves were generated using linearized plasmids with cloned fragments of the target genes in serial dilution. Melting curve analysis and agarose gel electrophoresis served to validate the amplification of the target genes. Primer sequences and thermal cycling conditions for all reactions are specified in Table S1.

### Statistical analysis

Statistical analyses were performed using the R software, version 4.3.3 (R Core Team 2024).

Treatment effects were determined by analyses of variance (ANOVA) using the *lm* and *anova* functions in the “stats” package. In phase 1, treatment and timepoint were included as separate factors, whereas in phase 2, treatment and timepoint were not independent and therefore had to be combined into a single factor. Variables not following a normal error distribution were Box-Cox transformed using the *boxcox* function in the “MASS” package (version 7.3–60.0.1, Venables and Ripley 2002) before analysis. Following significant overall effects (*P* < 0.05), post hoc pairwise comparisons were conducted using estimated marginal means with Bonferroni adjustment for multiple comparisons with the *emmeans* function in “emmeans”, version 1.10.6 (Lenth 2024).

## Results

### Phase 1: Soil herbicide content

In the 1 × treatment, clopyralid recovery decreased rapidly indicating high degradation rates and leading to a residual concentration below detection limit (< 0.050 mg kg^−1^) after 10 weeks (Fig. 1B). In the 10 × treatment, however, more than 50% of the initially added compound (1.433 ± 0.252 mg kg^−1^) was detected at the end of the experiment. The percentage metribuzin degradation followed the same pattern at both dose levels, indicating fast degradation even in the 10 × treatment, leaving 0.107 ± 0.006 and 1.567 ± 0.153 mg kg^−1^ (26% and 38%) three weeks after addition and 0.026 ± 0.001 and 0.477 ± 0.006 mg kg^−1^ (6% and 11%) 10 weeks after addition in the 1 × and 10 × treatment, respectively. Tembotrione could not be detected in the soil regardless of the dose and timepoint.

### Phase 1: Soil N pools and abundance of nitrifier groups

**Figure 2:**
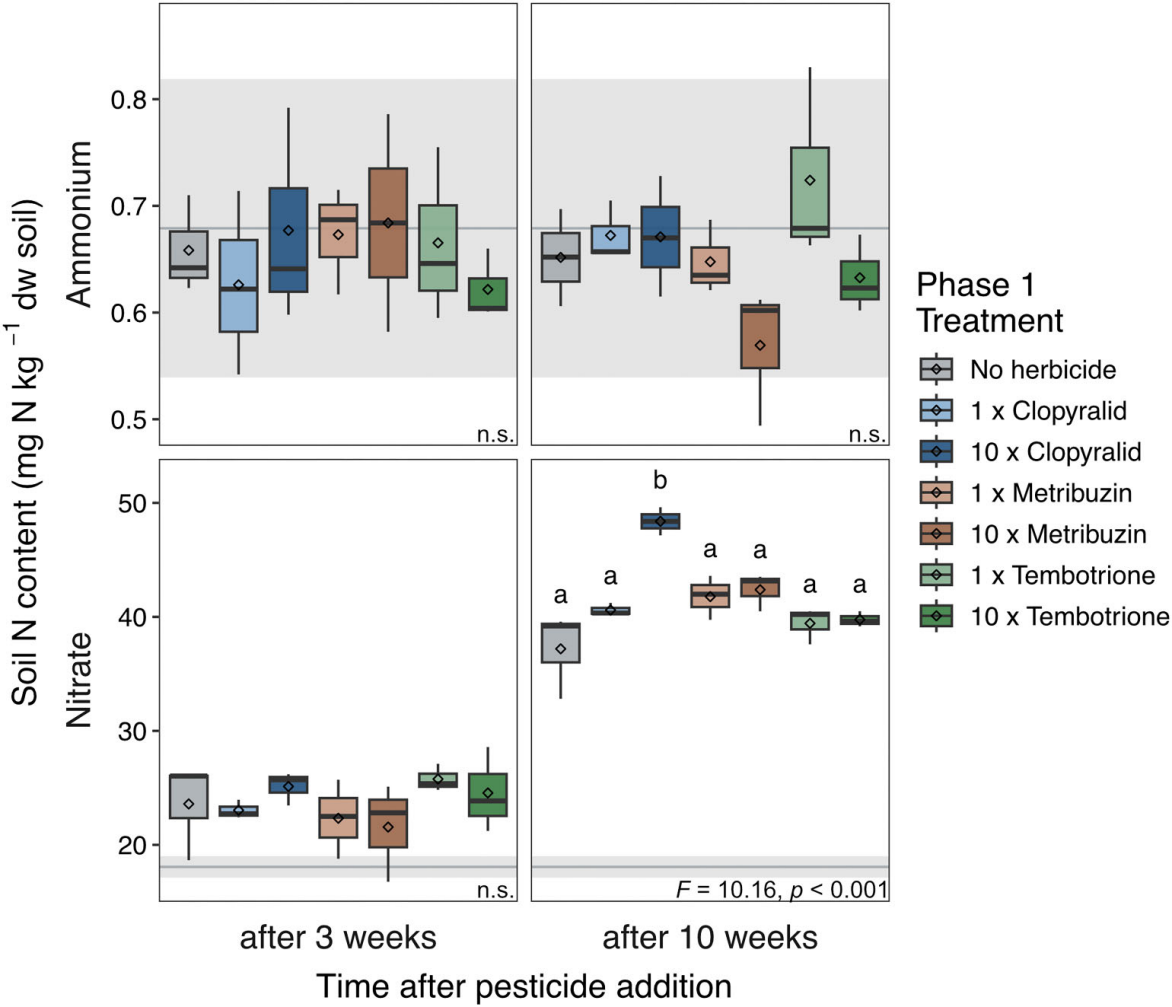
Ammonium and nitrate pools in the microcosms from phase 1, three and ten weeks after the addition of each herbicide at 1 × or 10 × the recommended dose and in the no-herbicide soil. Different letters above boxes within each panel indicate significant differences (ANOVA, *P*(F) < 0.05, followed by post hoc test, n = 3; n.s. not significant). Box boundaries represent first and third quartiles, with midline denoting the median, rhombus the average, and whiskers the 1.5 interquartile range. Horizontal grey line and shaded area indicate mean and standard deviation, respectively, of ammonium and nitrate content at the end of the acclimatization period of phase 1. Box colour indicates treatment.

The soil contained 0.68 ± 0.14 mg kg^−1^ DW ammonium-N (mean ± s.d.) at the end of the acclimatization period (day 7; Fig. 2). The ammonium content remained stable throughout the experiment in all treatments, with trends towards higher and lower ammonium in the 1 × tembotrione and 10 × metribuzin microcosms, respectively, after 10 weeks. By contrast, soil nitrate content increased over time, ranging from 18.07 ± 0.94 at the end of the acclimatization period to an average of 23.71 ± 2.96 at week 3, and 41.36 ± 3.71 mg kg^−1^ DW nitrate-N at week 10, indicating nitrification activity during the experiment. Only the 10 × clopyralid treatment affected nitrate levels, with an increase of 30% compared to the no-herbicide soil 10 weeks after addition.

**Figure 3:**
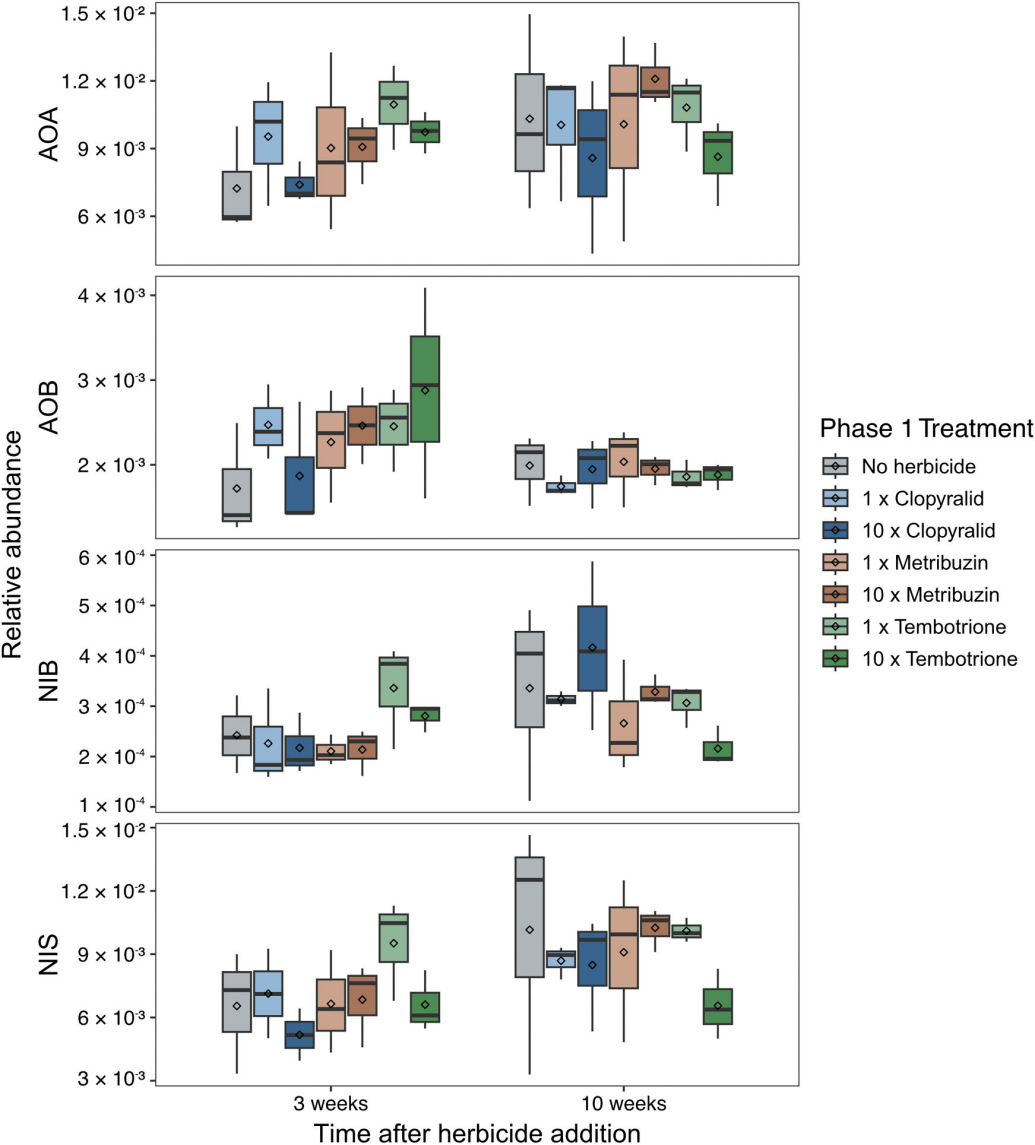
Relative abundance of nitrifying guilds in microcosms from phase 1 three and ten weeks after herbicide addition and in the no-herbicide soil, as measured by qPCR. Relative abundances were calculated as the ratio of nitrifier gene and 16S rRNA gene copy numbers for each guild: AOA (ammonia oxidizing archaea), AOB (ammonia oxidizing bacteria), NIB (*Nitrobacter* type nitrite oxidizers), NIS (*Nitrospira* type nitrite oxidizers). Box boundaries represent first and third quartiles, with midline denoting the median, rhombus the average, and whiskers the 1.5 interquartile range. No significant treatment effect was detected (ANOVA, *P*(F) > 0.05, n = 3). Box colours indicate treatment. Note the different scales on the y-axes.

Herbicide addition in phase 1 did not affect the relative abundance of nitrifiers, i.e. the ratio of nitrifier gene and 16S rRNA gene copy numbers (Fig. 3), nor their absolute abundance (Fig. S1) according to ANOVA (*P* > 0.05). However, there was a slight trend for higher relative abundance of AOA and AOB three weeks after herbicide addition compared to the no-herbicide soil, except in the 10 × clopyralid treatment. This was caused by a stronger decrease in the abundance of the total bacterial and archaeal community, measured by quantification of the 16S rRNA gene, in comparison with AOA or AOB abundance (Fig. S1). The relative abundance of NIB and NIS showed a decreased trend 10 weeks after the addition of 10 × tembotrione (Fig. 3), which was also reflected in the total abundance (Fig. S1).

### Phase 2: Soil N pools and abundance of nitrifier groups

**Figure 4:**
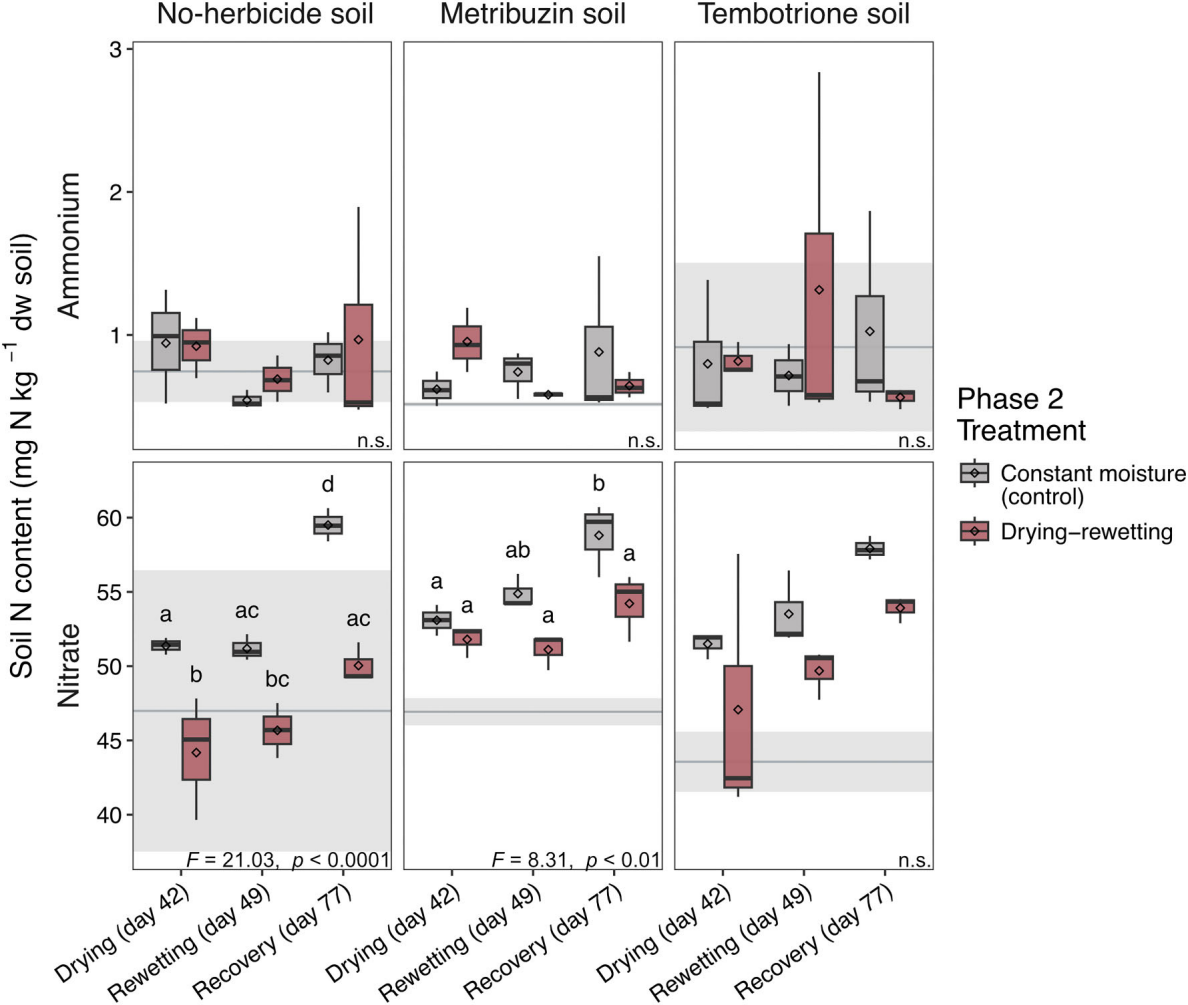
Ammonium and nitrate pools in the microcosms from phase 2 (no-herbicide, 10 × metribuzin and 10 × tembotrione soils from phase 1) during drying, after rewetting and at the end of the recovery period. Different letters above boxes within each panel indicate significant differences (ANOVA, *P*(F) < 0.05, followed by post hoc test, n = 3, n.s. not significant). Box boundaries represent first and third quartiles, with midline denoting the median, rhombus the average, and whiskers the 1.5 interquartile range. Horizontal grey line and shaded area indicate mean and standard deviation, respectively, of ammonium and nitrate content at the end of the acclimatization period of phase 2. Box colour indicates treatment.

Drying-rewetting did not affect soil ammonium content and, similar to phase 1, levels were stable over time albeit slightly higher (Figs. 2 and 4). Nitrate levels again increased over time, however this increase was smaller in drought-treated soils compared to the controls kept at constant moisture, with levels increasing by 4% and 13% at the end of the drought period, 7% and 16% after rewetting, and 15% and 28% at the end of the experiment in drought-treated and control soils, respectively. The effect of drying-rewetting on nitrate levels was dependent on the herbicide addition in phase 1. In the no-herbicide soil, nitrate levels were significantly lower in dry soil compared to nitrate levels in the control. In the metribuzin soil, the difference between control and drought-treated soils increased over time and differed significantly first after the recovery period due to the increased nitrate level in the control. In the tembotrione soil, there was no overall effect of drying-rewetting on nitrate content, although a trend of lower nitrate levels was observed in the drought-treated soil, similar to the other two soils from phase 1.

**Figure 5:**
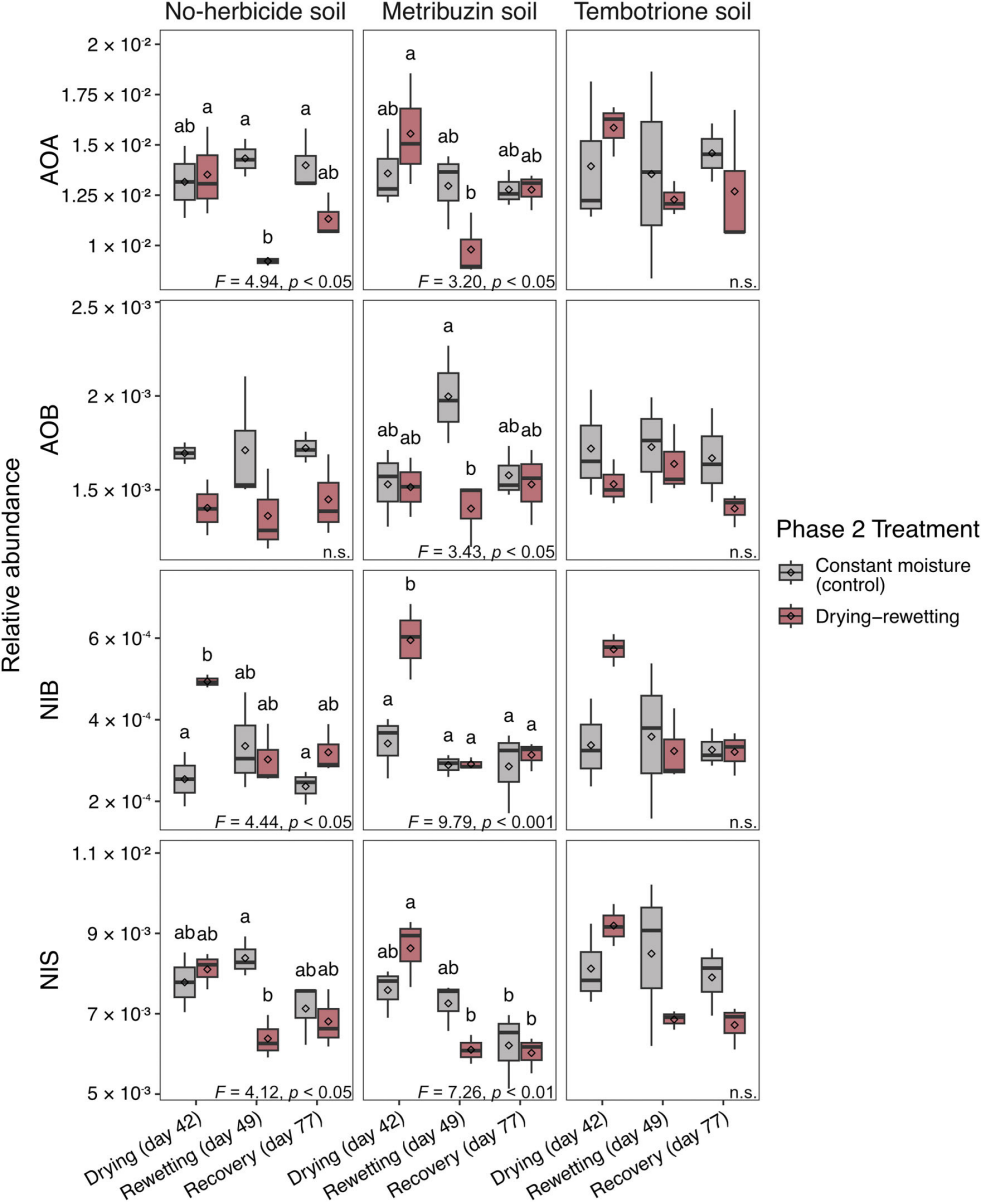
Relative abundance of nitrifying guilds in the microcosms from phase 2, during drying, after rewetting and at the end of the recovery period. Relative abundances were calculated as the ratio of nitrifier gene and 16S rRNA gene copy numbers for each guild: AOA (ammonia oxidizing archaea), AOB (ammonia oxidizing bacteria), NIB (*Nitrobacter* type nitrite oxidizers), NIS (*Nitrospira* type nitrite oxidizers). Different letters above boxes within each panel indicate significant differences (ANOVA, *p*(F) < 0.05, followed by post hoc test, n = 3, n.s. not significant). Box boundaries represent first and third quartiles, with midline denoting the median, rhombus the average, and whiskers the 1.5 interquartile range. Box colours indicate treatment. Note the different scales on the *y*-axes.

In contrast to herbicide addition, drying-rewetting significantly affected abundances of the four nitrifier guilds (Fig. 5). In the no-herbicide soil, the relative abundance of NIB increased significantly during drying in comparison to that of the control but returned to the same level after rewetting, while AOA and NIS significantly decreased after rewetting and AOB were not affected. In the metribuzin soil, all four guilds were significantly affected by drought, with all but the AOB increasing during drying and decreasing after rewetting. After the recovery period, relative abundances resembled those of the soil kept at constant moisture. In the tembotrione soil, there was no significant effect of drying-rewetting on relative abundances, but similar trends were observed as in the no-herbicide and metribuzin soils. No interaction effect was found between herbicide exposure in phase 1 and the combination of drought treatment and timepoint in phase 2 (ANOVA, *P* > 0.05, Table S2).

The absolute abundances of the nitrifier guilds and total prokaryotic community in the no-herbicide soil were all significantly higher in the droughted soil than in the control at the first time point (“drying”), but this difference was no longer apparent from the rewetting onwards (Fig. S2). A similar pattern was visible in the metribuzin and tembotrione soils, but in the latter, significant effects of drying-rewetting were only observed for AOB and NIB.

## Discussion

The addition of clopyralid, metribuzin, or tembotrione, regardless the dose, did not affect the absolute or relative abundance of nitrifying guilds and showed no inhibitory effects on nitrification activity based on soil nitrate pools, when not subject to an additional stressor. We even observed an increase in nitrification activity with the high dose of clopyralid. The compound itself cannot be the source of N for this increase, as the added amount contained 0.145 mg N kg^–1^ dw soil, while nitrate increased by almost 10 mg N kg^–1^ dw soil in comparison to the no-herbicide soil. Instead, clopyralid might have served as a carbon source for micro-organisms (Karpouzas et al. 2016) and primed N mineralization, thereby indirectly affecting nitrate levels. The lack of negative effects of the added herbicides are consistent with those from assays using single nitrifier species, which demonstrated that these herbicides had no effect at 1 × and 10 × of the recommended dose on the most sensitive AOA, AOB, and NIB strains, with the exception of the addition of 10 × metribuzin that caused partial inhibition of ammonia oxidation in AOA (Bachtsevani 2024). Our findings also align with recent reports of no effects on the abundance of AOA and AOB in soil microcosm studies conducted under controlled conditions using clopyralid in formulation (Sim et al. 2022) or as a pure compound (Drocco et al. 2025). Moreover, clopyralid in formulation did not impact the overall bacterial community composition in field experiments (Tomco et al. 2016). By mimicking auxin, a hormone promoting growth and development in plants (Vanneste and Friml 2009), clopyralid triggers excessive tissue growth leading to the plant’s death. Auxin is also known to be involved in interactions between plants and plant growth-promoting rhizobacteria as a signalling molecule, where these micro-organisms also produce auxin (Spaepen and Vanderleyden 2011). This suggests that auxin itself is unlikely to directly inhibit micro-organisms. Under field conditions however, herbicide effects may propagate through plant-microbe interactions, as altered plant physiology can modify root exudates, which influence microbial activity and community structure (de Vries et al. 2020, Ruuskanen et al. 2023). Studies on effects of metribuzin on soil micro-organisms are scarce and mainly limited to the microbial impact on the degradation of this herbicide. However, no effect of metribuzin as a pure compound on soil respiration or decomposition rates (Lewis et al. 1978) or on nitrification activity in field experiments spanning various soil types (Junnila et al. 1993) has been reported, which is consistent with our results. Tembotrione and other structurally similar beta-triketones are HPPD inhibitors leading to bleaching of foliage and consequently plant death (Moran 2005, Dayan et al. 2007). Even though HPPD is found in micro-organisms, including AOA (Herbold et al. 2017) and AOB (Rice et al. 2016), the impact of these inhibitory compounds on soil microbial communities seems to be limited (Thiour-Mauprivez et al. 2021), which agrees with our observations.

In contrast to herbicide addition, drying-rewetting as a single stressor (i.e. in no-herbicide soil) affected absolute and relative abundances of most of the nitrifying guilds as well as the nitrate pools. The temporary increases in absolute abundance in the no-herbicide soil subject to drought followed by the return to the same levels as in the constantly moist control suggest resilient nitrifier guilds. However, the relative abundance reveals a more nuanced picture of their response to drying-rewetting. In line with our second hypothesis, relative abundances show differing responses between the functional guilds, with generally low resistance to rewetting in AOA and NIS and a tendency for low resilience in the AOA community. Rewetting is characterized by a drop in osmolality and an increase in soil ammonium, nitrate, and carbon compounds following resuspension and cell death (Birch 1958). Considering the overall increase in bacterial and archaeal abundance during drying, the relative decrease in AOA and NIS after rewetting could be caused by a lower capacity to rapidly re-adjust osmolality by disposing of salts and compatible solutes upon rewetting (Halverson et al. 2000) and hence cell lysis (Bottner 1985). The differences observed between functional guilds may also be influenced by higher substrate levels, which can pose a disadvantage on AOA and NIS due to, on average, higher substrate affinity compared to AOB and NIB, respectively (Thion and Prosser 2014, Simonin et al. 2015, Sterngren et al. 2015). This agrees with previous studies reporting AOB to be less sensitive than AOA to drought in both microcosm (Thion and Prosser 2014, Bello et al. 2019, Müller et al. 2025) and field experiments (Fuchslueger et al. 2014, Séneca et al. 2020).

Contrary to our third hypothesis, the drought effects on the relative abundance of nitrifier guilds were similar in soil previously treated with metribuzin compared to the no-herbicide soil, while there was no overall effect of drying-rewetting in the tembotrione-treated soil. This could indicate different responses of nitrifying guilds to drying-rewetting between metribuzin- and tembotrione-treated soils, although the trends were similar across all treatments. Interestingly, the difference in nitrate production between soils subject to drying-rewetting and constantly moist soils appeared smaller in the herbicide-treated soils than in the no-herbicide soil. However, this pattern was not statistically supported and should therefore be interpreted with caution. Herbicide exposure may have altered microbial community composition or activity in ways that modified subsequent responses to drying–rewetting. In this context, the concept of species’ co-tolerance offers a possible interpretation, stating that a first disturbance—in this study herbicide addition—can increase the resistance to a second disturbance—the drying-rewetting cycles—depending on the disturbance’s mechanism of action (Vinebrooke et al. 2004, Renes et al. 2020). This would imply an adaptation not captured by solely assessing abundances of the functional guilds, however these interpretations remain speculative.

Suggestions have been made to improve risk assessment procedures at both pre- and post-authorization steps (Storck et al. 2017, Möhring et al. 2020), including the quantification of functional genes (Wessén and Hallin 2011, Karpouzas et al. 2022). While functional gene quantification could be a suitable tool for assessing toxic effects exceeding a certain threshold, this work shows that the evaluation of multiple stressors in environmental risk assessment requires combining effects on ecological processes, such as resistance and resilience, with methods reaching beyond DNA-based quantification of microbial groups. Considering multiple stressors in environmental risk assessment allows for the refinement of the traditional approach of using safety (or uncertainty) factors to account for knowledge gaps (Chapman et al. 1998). Moreover, standardized toxicity tests are usually performed under optimal conditions, but with a range of possible additional biotic and abiotic stressors, the uncertainty in environmental risk assessment is high. Our results demonstrate that in this sandy soil, drying-rewetting is a much stronger stressor than exposure to one of the three tested herbicides, however differences in drought response depending on herbicide exposure could not be proven with the available data. Given the generally low effect of the tested herbicides in this and previous work, this is not surprising. Nevertheless, the sensitivity of ammonia and nitrite oxidizing guilds to drought stress highlights their potential suitability as microbial indicators for risk assessments in soil, and future research aiming to improve risk assessment procedures should evaluate interactive effects between pesticides and additional stressors as well as determine effects on nitrifying community composition, including comammox bacteria.

## Supplementary Material

fiag038_Supplemental_Files

## Data Availability

All data can be found in Supplementary material.
