# Supplementary material for: Legacy effects of herbicides on soil nitrifying guilds exposed to drought
Source: FEMS Microbiol Ecol. 2026 Apr 16;102(5):fiag038. doi: 10.1093/femsec/fiag038 (PMC13131219; doi:10.1093/femsec/fiag038)
Supplement: fiag038_Supplemental_Files [file fiag038_supplemental_files.zip › Muller_supplementary_1_revised.docx]

– SUPPLEMENTARY INFORMATION –

**Legacy effects of herbicides on soil nitrifying guilds exposed to drought**

Laura J. Müller, Aurélien Saghaï, Christopher Jones, Sara Hallin

**Content**

| **Table S1** | Primers and thermal cycling conditions for the quantitative PCRs |
| --- | --- |
| **Table S2** | F-values obtained from two-way ANOVA of values collected in phase 2 |
| **Figure S1** | Absolute abundance of genes reflecting the total prokaryotic community and nitrifying guilds in phase 1 (herbicide addition) |
| **Figure S2** | Absolute abundance of genes reflecting the total prokaryotic community and nitrifying guilds in phase 2 (drying-rewetting) |

**Table S1.** Primers and thermal cycling conditions for the quantitative PCRs. Melting curve conditions are added in italic.

| **Group** | **Target (gene)** | **Primer** | **Sequence (5’–3’)** | **Reference** | **Conc.** | **PCR conditions** |
| --- | --- | --- | --- | --- | --- | --- |
| Prokaryotes | 16 rRNA | 515 F Parada | GTGYCAGCMGCCGCGGTAA | (Parada et al. 2016) | 0.5 µM | 95°C 5 min;  (95°C 15s, 50°C 30s, 72°C 30s, 78°C 5s) x 34  *95°C 10s, 60 °C – 95 °C (increment 0.5 °C 5 s^-1^)* |
|  |  | 926 R Quince | CCGYCAATTYMTTTRAGTTT | (Quince et al. 2011) |  |  |
| AOA | archaeal *amoA* | crenamoA23F | ATGGTCTGGCTWAGACG | (Tourna et al. 2008) | 0.5 µM | 95°C 5 min;  (95°C 15s, 55°C 30s, 72°C 40s) x 40  *95°C 15s, 60 °C – 95 °C (increment 0.5 °C s^-1^)* |
|  |  | crenamoA616R | GCCATCCATCTGTATGTCCA |  |  |  |
| AOB | bacterial *amoA* | AmoA1F | GGGGTTTCTACTGGTGGT | (Rotthauwe et al. 1997) | 0.5 µM | 95°C 3 min;  (95°C 15s, 55°C 30s, 72°C 40s, 80°C 20s) x 40  *95°C 15s, 60 °C – 95 °C (increment 0.5 °C s^-1^)* |
|  |  | AmoA2R | CCCCTCKGSAAAGCCTTCTTC |  |  |  |
| NIB | *Nitrobacter* type *nxrB* | nxrB1F | ACGTGGAGACCAAGCCGGG | (Vanparys et al. 2007) | 0.5 µM | 95°C 3 min;  (95°C 15s, 72–67°C 30s, 72°C 30s, 78°C 20s) x 6;  (95°C 15s, 67°C 30s, 72°C 30s, 78°C 20s) x 29;  95°C 20s  *65 °C – 95 °C (increment 0.5 °C 5 s^-1^)* |
|  |  | nxrB1R | CCGTGCTGTTGAYCTCGTTGA |  |  |  |
| NIS | *Nitrospira* type *nxrB* | nxrB169f | TACATGTGGTGGAACA | (Pester et al. 2014) | 0.5 µM | 95°C 3 min;  (95°C 15s, 56°C 30s, 72°C 45s, 78°C 20s) x 35;  95°C 15s  *65 °C – 95 °C (increment 0.5 °C 5 s^-1^)* |
|  |  | nxrB638r | CGGTTCTGGTCRATCA |  |  |  |

**Table S2.** F-values obtained from two-way ANOVA of values collected in phase 2 of the experiment. The analysis included the pesticide exposure treatment from phase 1, a combined factor of the drought treatment and timepoint from phase 2 (combined due to covariance), and their interaction. Statistical significance is indicated by asterisks (^***^*p* < 0.001, ^**^0.001 < *p* < 0.01, ^*^0.01 < *p ≤* 0.05).

|  | **Treatment phase 1** | **Treatment and timepoint phase 2**  **(combined factor)** | **Treatment and timepoint phase 1 x**  **Treatment phase 2** |
| --- | --- | --- | --- |
| **NH_4_^+^-N** | 0.055 | 1.447 | 0.472 |
| **NO_3_^–^-N** | 8.000** | 18.115*** | 1.043 |
| **Archaeal *amoA* abundance** | 2.559 | 9.880*** | 0.232 |
| **Bacterial *amoA* abundance** | 5.559** | 14.956*** | 0.504 |
| ***Nitrobacter-*type *nxrB* abundance** | 1.555 | 26.120*** | 0.525 |
| ***Nitrospira-*type *nxrB* abundance** | 5.292** | 15.845*** | 0.461 |
| **16S abundance** | 3.461* | 13.220*** | 0.813 |
| **Archaeal *amoA* rel. abundance** | 1.421 | 4.377** | 0.468 |
| **Bacterial *amoA* rel. abundance** | 0.4 | 4.184** | 0.939 |
| ***Nitrobacter-*type *nxrB* rel. abundance** | 1.434 | 13.266*** | 0.433 |
| ***Nitrospira-*type *nxrB* rel. abundance** | 5.579** | 10.488*** | 0.568 |


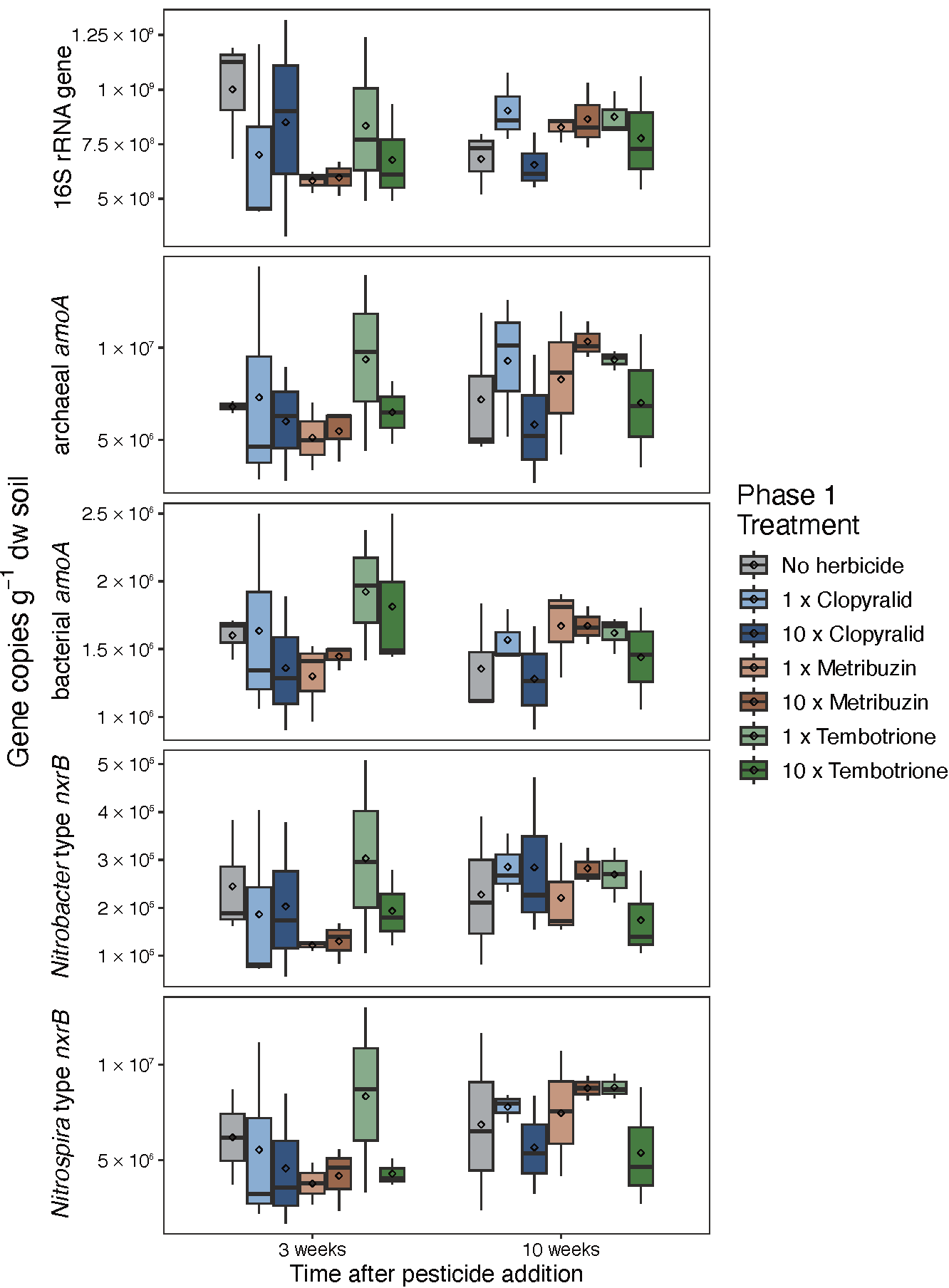


**Fig. S1.** Absolute abundance of genes reflecting the total prokaryotic community and nitrifying guilds in phase 1, three weeks and 10 weeks after herbicide addition and in the uncontaminated soil. 16S rRNA gene (total prokaryotic community), archaeal *amoA* (AOA, ammonia oxidizing archaea), bacterial *amoA* (AOB, ammonia oxidizing bacteria), *Nitrobacter*-type *nxrB* (NIB, *Nitrobacter*-type nitrite oxidizing bacteria), *Nitrospira*-type *nxrB* (NIS, *Nitrospira*-type nitrite oxidizing bacteria). Box boundaries represent first and third quartiles, with midline denoting the median, rhombus the average, and whiskers the 1.5 interquartile range. No significant treatment effect was detected using ANOVA with a significance level of *p* = 0.05. Box colours indicate treatment.


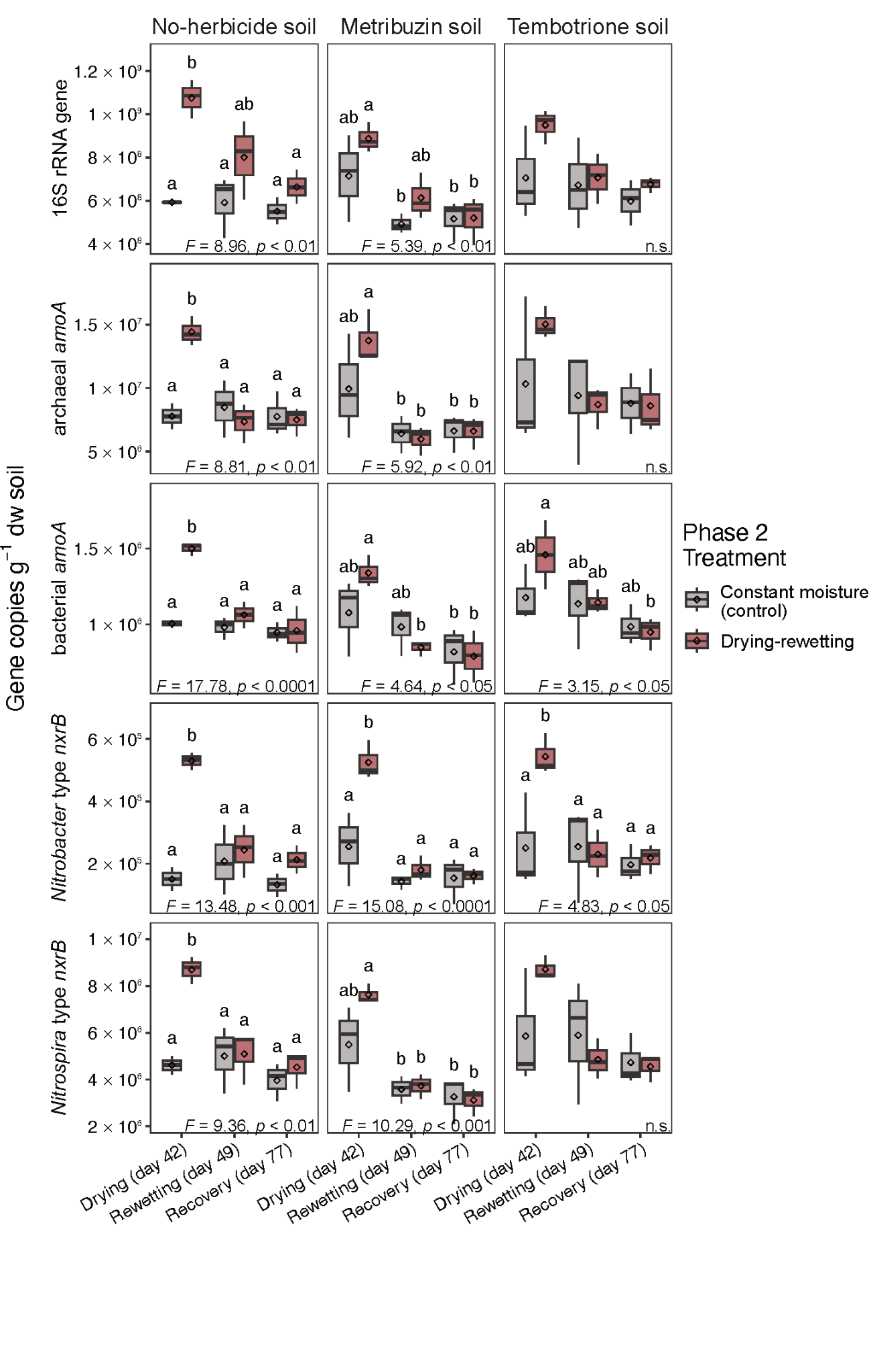


**Fig. S2.** Absolute abundance of genes reflecting the total prokaryotic community and nitrifying guilds in phase 2 during drought, after rewetting and after the recovery period. 16S rRNA gene (total prokaryotic community), bacterial *amoA* (AOB, ammonia oxidizing bacteria), *Nitrobacter*-type *nxrB* (NIB, *Nitrobacter*-type nitrite oxidizing bacteria), *Nitrospira*-type *nxrB* (NIS, *Nitrospira*-type nitrite oxidizing bacteria). Different letters above boxes within each indicate significant differences according to post hoc tests after significant overall effects according to ANOVA (*p* < 0.05, n = 3, n.s. not significant). Box boundaries represent first and third quartiles, with midline denoting the median, rhombus the average, and whiskers the 1.5 interquartile range. Box colours indicate treatment.

**References**

Parada, A.E., Needham, D.M. & Fuhrman, J.A. (2016). Every base matters: assessing small subunit rRNA primers for marine microbiomes with mock communities, time series and global field samples. *Environmental Microbiology*, 18 (5), 1403–1414. https://doi.org/10.1111/1462-2920.13023

Pester, M., Maixner, F., Berry, D., Rattei, T., Koch, H., Lücker, S., Nowka, B., Richter, A., Spieck, E., Lebedeva, E., Loy, A., Wagner, M. & Daims, H. (2014). *NxrB* encoding the beta subunit of nitrite oxidoreductase as functional and phylogenetic marker for nitrite-oxidizing *Nitrospira*. *Environmental Microbiology*, 16 (10), 3055–3071. https://doi.org/10.1111/1462-2920.12300

Quince, C., Lanzen, A., Davenport, R.J. & Turnbaugh, P.J. (2011). Removing Noise From Pyrosequenced Amplicons. *BMC Bioinformatics*, 12 (1), 38. https://doi.org/10.1186/1471-2105-12-38

Rotthauwe, J.H., Witzel, K.P. & Liesack, W. (1997). The ammonia monooxygenase structural gene *amoA* as a functional marker: molecular fine-scale analysis of natural ammonia-oxidizing populations. *Applied and Environmental Microbiology*, 63 (12), 4704–4712. https://doi.org/10.1128/aem.63.12.4704-4712.1997

Tourna, M., Freitag, T.E., Nicol, G.W. & Prosser, J.I. (2008). Growth, activity and temperature responses of ammonia‐oxidizing archaea and bacteria in soil microcosms. *Environmental Microbiology*, 10 (5), 1357–1364

Vanparys, B., Spieck, E., Heylen, K., Wittebolle, L., Geets, J., Boon, N. & De Vos, P. (2007). The phylogeny of the genus *Nitrobacter* based on comparative rep-PCR, 16S rRNA and nitrite oxidoreductase gene sequence analysis. *Systematic and Applied Microbiology*, 30 (4), 297–308. https://doi.org/10.1016/j.syapm.2006.11.006
